# Elucidating the contributions of multiple aldehyde/alcohol dehydrogenases to butanol and ethanol production in *Clostridium acetobutylicum*

**DOI:** 10.1038/srep28189

**Published:** 2016-06-20

**Authors:** Zongjie Dai, Hongjun Dong, Yanping Zhang, Yin Li

**Affiliations:** 1 CAS Key Laboratory of Microbial Physiological and Metabolic Engineering, Institute of Microbiology, Chinese Academy of Sciences, Beijing, 100101, China; 2 Department of Biochemistry and Molecular Biology, University of Science and Technology of China, Hefei, 230026, China

## Abstract

Ethanol and butanol biosynthesis in *Clostridium acetobutylicum* share common aldehyde/alcohol dehydrogenases. However, little is known about the relative contributions of these multiple dehydrogenases to ethanol and butanol production respectively. The contributions of six aldehyde/alcohol dehydrogenases of *C. acetobutylicum* on butanol and ethanol production were evaluated through inactivation of the corresponding genes respectively. For butanol production, the relative contributions from these enzymes were: AdhE1 > BdhB > BdhA ≈ YqhD > SMB_P058 > AdhE2. For ethanol production, the contributions were: AdhE1 > BdhB > YqhD > SMB_P058 > AdhE2 > BdhA. AdhE1 and BdhB are two essential enzymes for butanol and ethanol production. AdhE1 was relatively specific for butanol production over ethanol, while BdhB, YqhD, and SMB_P058 favor ethanol production over butanol. Butanol synthesis was increased in the *adhE2* mutant, which had a higher butanol/ethanol ratio (8.15:1) compared with wild type strain (6.65:1). Both the *SMB_P058* mutant and *yqhD* mutant produced less ethanol without loss of butanol formation, which led to higher butanol/ethanol ratio, 10.12:1 and 10.17:1, respectively. To engineer a more efficient butanol-producing strain, *adhE1* could be overexpressed, furthermore, *adhE2*, *SMB_P058*, *yqhD* are promising gene inactivation targets. This work provides useful information guiding future strain improvement for butanol production.

Microbial production of chemicals and fuels from renewable resources is a prospective approach to avoid problems that are usually associated with fossil-based chemical synthesis, such as environmental pollution, unsustainable demand, and increasing costs. Alcohols, such as 1,3-propanediol, butanol, isobutanol, and 1,4-butanediol can be used as important platform chemicals or biofuels. Many efforts have been made to improve production of such target alcohols by engineering aldehyde/alcohol dehydrogenases, as they are commonly the final enzymes used for converting acyl-CoAs or aldehydes to their corresponding alcohols.

The substrate specificity of the aldehyde/alcohol dehydrogenase is critical for the production of target alcohol. Among the multiple alcohol dehydrogenases of *Escherichia coli*, it was found that the endogenous alcohol dehydrogenase YqhD contributed most to isobutanol production. Exogenous enzymes have also been utilized to increase alcohol production. When exogenous AdhA from *Lactococcus lactis* was expressed in *E. coli*, it led to better isobutanol production due to its higher isobutyraldehyde reductase activity than the native enzymes including YqhD^1^. Expression of AdhE2 from *C. acetobutylicum* was also more optimal for production of butanol than the native AdhE of *E. coli* as a result of this enzyme’s higher affinity for butyryl-CoA and lower affinity for acetyl-CoA. Researchers therefore engineered the strain for optimal butanol production by deleting *E. coli adhE* and expressing the exogenous *adhE2*^2^,^3^. The same engineering strategy was also successfully used for 1,4-butanediol production in *E. coli*^4^. Notably, in an attempt to obtain an engineered *E. coli* strain capable of producing a high-titer of 1,3-propanediol, the broad-spectrum hypothetical dehydrogenase (encoded by *yqhD*) from *E. coli* showed better performance than the specific 1,3-propanediol dehydrogenase (encoded by *dhaT*) from *Klebsiella pneumoniae*^5^, indicating that exogenous dehydrogenases might not always be the optimal enzymes for target production. In fact, there are many aldehyde/alcohol dehydrogenases in microbial cells, but their physiological functions are not always clear and their relative contributions to alcohols production are unknown. In some species, different aldehyde/alcohol dehydrogenases contribute to the production of one alcohol while in other species, the same aldehyde/alcohol dehydrogenase(s) might contribute to the production of different alcohols.

In *C. acetobutylicum*, a typical butanol-producing microorganism, there are many aldehyde/alcohol dehydrogenases for alcohol production. Besides the four known dehydrogenases (biofunctional aldehyde/alcohol dehydrogenase: AdhE1 and AdhE2^6^,^7^, alcohol dehydrogenases: BdhA and BdhB^8^), there are other genes annotated as encoding alcohol dehydrogenases in the genomes of *C. acetobutylicum* ATCC 824^9^ and *C. acetobutylicum* DSM 1731^10^, including *cap0059* (*SMB_P058*), *cac3392 (yqhD)*, *cac3484*, and *cac3375*. The presence of so many aldehyde/alcohol dehydrogenases increases the complexity of engineering a higher (or homo-) butanol producing strain. The production of butanol is always accompanied by the production of ethanol, a phenomenon that may be due to the low specificity of the aldehyde/alcohol dehydrogenases for their substrate (butyryl-CoA and butyraldehyde). As a consequence, by-product ethanol concentrations often increased as butanol production increases. For example, in a *C. acetobutylicum solR* gene knockout strain, overexpression of the bifunctional alcohol/aldehyde dehydrogenase gene *adhE1* increased butanol production by 21%, however this increased ethanol production also by 62%^11^. Similarly when the *adhE1*^*D485G*^gene was overexpressed in a *pta*-*buk* double deficient *C. acetobutylicum* strain, butanol production increased by 160% compared with wild-type, however, ethanol production also increased by 233%^12^. This phenomenon does not only occur in *C. acetobutylicum* engineered for butanol production; when *E. coli* are engineered for butanol biosynthesis via the classic clostridial fermentation pathway or reverse *β*-oxidation pathway^3^,^13^, ethanol is also produced as by-product. Furthermore, the overproduction of aldehyde/alcohol dehydrogenases does not always increase butanol and ethanol by the same relative amounts. Each dehydrogenase contributes differently to butanol or ethanol production, and their specific contributions have, so far, not been characterized.

According to transcriptomic results for *C. acetobutylicum* ATCC 824, *cap0059* (*SMB_P058*), which encodes for an alcohol dehydrogenase, is transcribed as well as *adhE1*, *adhE2*, *bdhA* and *bdhB*^14^. In a previous proteomic analysis, we found that a broad-spectrum hypothetical alcohol dehydrogenase YqhD was highly expressed and AdhE1, BdhA and BdhB were also detected^15^. In this study, we therefore aimed to determine the contributions of each of these six aldehyde/alcohol dehydrogenase in *C. acetobutylicum* DSM 1731. The six genes were individually disrupted, and their physiological functions and specific contributions to butanol and ethanol synthesis were investigated.

## Results

### Generation of aldehyde/alcohol dehydrogenases disruption mutants in *C. acetobutylicum* DSM 1731

**Figure 1:**
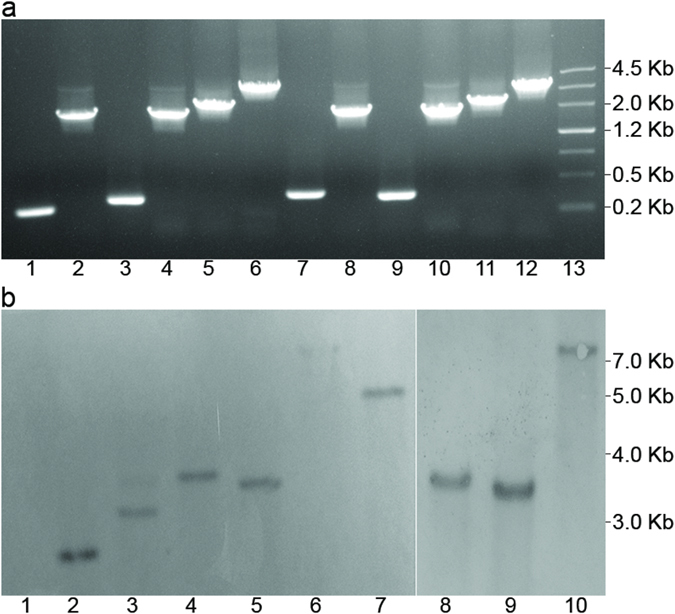
Construction of different aldehyde/alcohol dehydrogenases mutants of *C. acetobutylicum* DSM 1731. (**a**) Identification of the mutants by PCR using primers flanking the insertion site. Lane 1, 3, 5, 7, 9, 11, wild-type; lane 2, *bdhA* mutant; lane 4, *bdhB* mutant; lane 6, *adhE1* mutant; lane 8, *yqhD* mutant; lane 10, *SMB_P058* mutant; lane 12, *adhE2* mutant and lane 13, maker. (**b**) Southern blot confirmation of intron insertion into the aldehyde/alcohol dehydrogenase genes using the intron specific probe 1, wild-type; 2, *SMB_P058* mutant; 3, *yqhD* mutant; 4, *bdhB* mutant; 5, *bdhA* mutant; 6, *adhE2* mutant (weak band); 7, *adhE1* mutant; 8, *bdhB* mutant (repeated as reference for *adhE2* mutant); 9, *bdhA* mutant (repeated as reference for the *adhE2* mutant); 10, *adhE2* mutant (repeated).

To characterize the contribution of multiple aldehyde/alcohol dehydrogenases in butanol and ethanol production in *C. acetobutylicum*, the ClosTron system^16^ with slight modifications^17^ was used to create aldehyde/alcohol dehydrogenase negative mutants. According to the genome information of *C. acetobutylicum* DSM 1731^10^, the insertion sites for alcohol dehydrogenase genes: *adhE1*, *adhE2*, *bdhA*, *bdhB, SMB_P058*, and *yqhD* were calculated by submitting the corresponding gene sequence into a computer algorithm^18^. The insertion sites and intron re-targeting PCR primers are described in Additional File1: Table S1. Putative erythromycin-resistant transformants were identified by PCR screening (see Methods). Compared with the specific PCR products from wild-type genes, the mutants yielded enlarged fragments (Fig. 1a). Southern blot hybridization was performed to verify the genomic structure of the mutants. An intron specific probe was used to hybridize the expected fragment. Bands with the expected sizes were detected on the hybridization membrane (Fig. 1b). Positive clones of the alcohol dehydrogenases negative mutants were designated *C. acetobutylicum* DSM 1731 *adhE1*::int(807), *C. acetobutylicum* DSM 1731 *adhE2*::int(846), *C. acetobutylicum* DSM 1731 *bdhA*::int(133), *C. acetobutylicum* DSM 1731 *bdhB*::int(669), *C. acetobutylicum* DSM 1731 *SMB_P058*::int(150) and *C. acetobutylicum* DSM 1731 *yqhD*::int(405), and were used for further studies.

### Disruption of *adhE1* results in loss of ethanol and butanol production however *adhE2* disruption increases butanol production in *C. acetobutylicum*

AdhE1 and AdhE2 are two bifunctional dehydrogenases, which convert acyl-CoA to aldehyde then to alcohol in two reductive steps using NADH as cofactor^6^,^7^. However, their contributions for ethanol and butanol production is different.

**Figure 2:**
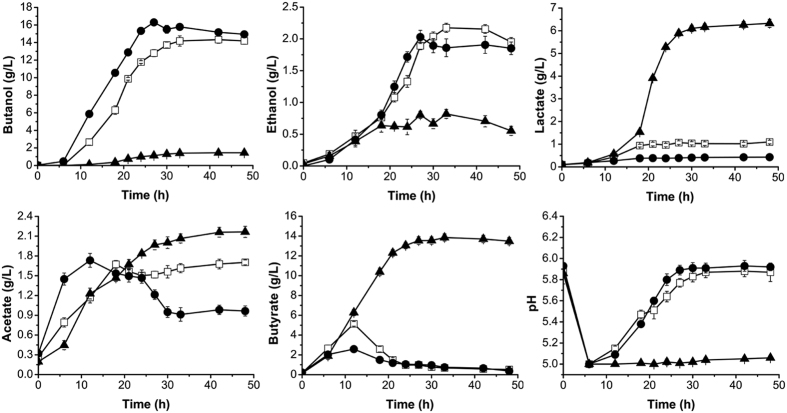
Fermentation profiles of *C. acetobutylicum* DSM 1731, *adhE1* mutant and *adhE2* mutant. White squares, wild-type; black triangles, *adhE1* mutant; black circles, *adhE2* mutant.

Compared with the wild-type strain, mutant *C. acetobutylicum* DSM 1731 *adhE1*::int(807) almost lost the ability to produce alcohol with only minor ethanol (0.6 g/L) and butanol (1.5 g/L) production after 48 h fermentation (Fig. 2). Interestingly, *C. acetobutylicum* DSM 1731 *adhE2*::int(846) showed a highly different metabolic profile compared with the *adhE1* mutant. The *adhE2* mutant reached a butanol concentration of 16.3 g/L, an increase of 12.5% compared with the control strain. Meanwhile, the ethanol concentration decreased to 2.0 g/L (Fig. 2), thus there was an increase in the butanol/ethanol ratio from 6.65:1 for the wild-type strain to 8.15:1 for the *adhE2* mutant. This mutant consumed 80 g/L glucose after 48 h fermentation with a solvent yield of 31.8% and a butanol yield of 20.3%, both of which were higher than for the wild-type strain (Table 1). Furthermore, the production of another NADH dependent-acid lactate was increased, to 6.3 g/L in *adhE1* mutant, compared with 1.0 g/L produced by the control strain, while less lactate (0.2 g/L) accumulated in *adhE2* mutant culture medium (Fig. 2).

As to acid production, the *adhE1* mutant strain showed a sustained increase in acid accumulation producing 2.2 g/L acetate and 13.6 g/L butyrate compared with 1.7 g/L acetate and 0.5 g/L butyrate for the wild-type strain which re-assimilates acid. The residual acetate decreased to 0.5 g/L in the *adhE2* mutant, and there was no remarkable difference in the concentration of residual butyrate between the *adhE2* mutant and the wild-type strain, but the peak level of butyrate was much lower for the mutant than the control (Fig. 2). These results indicate that the disruption of *adhE1* leads to a failed conversion from acidogenesis to solventogenesis and the ability to re-assimilate acid, while the disruption of *adhE2* increases the bacterium’s ability to produce solvent. As the result, there was no visible rebound of the pH value during the fermentation process of the *adhE1* mutant strain, due to the lessened acid re-assimilation of this strain. Contrastingly, the pH values of the control strain and the *adhE2* mutant rebounded. This can be seen with the final pH of *adhE2* mutant being 5.93, a higher value than that of the control (pH 5.88) (Fig. 2).

### BdhB is more important for alcohol production than BdhA in *C. acetobutylicum*

**Figure 3:**
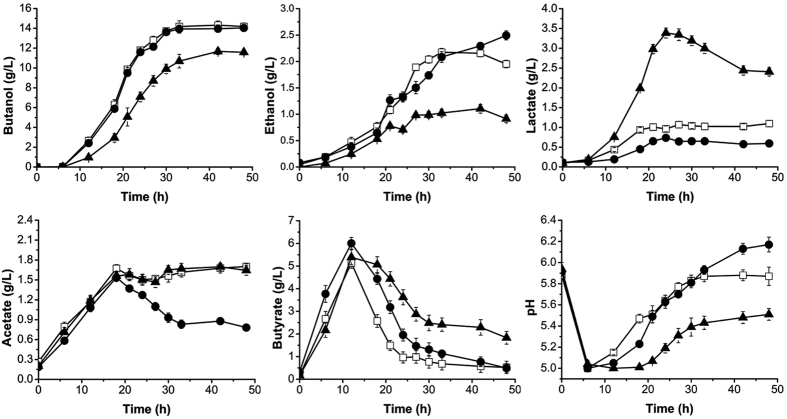
Fermentation profiles of *C. acetobutylicum* DSM 1731, *bdhA* mutant and *bdhB* mutant. White squares, wild-type; black triangles, *bdhB* mutant; black circles, *bdhA* mutant.

BdhA and BdhB are two NAD(P)H dependent alcohol dehydrogenases that convert aldehydes to alcohols^8^,^19^. After 48 h fermentation, the mutant strain *C. acetobutylicum* DSM 1731 *bdhA*::int(133) produced similar amounts of butanol to the control strain, however, it also produced more ethanol (2.49 g/L) than the control (Fig. 3) with an overall butanol/ethanol ratio lower than the control (5.63:1 vs 6.65:1). This mutant strain also consumed less glucose (74 g/L) than the control (78 g/L) (Fig. 3), leading to a higher solvent (31.5%) and butanol yield (19.0%) compared with the control (Table 1). However, after disrupting *bdhB* (*SMB_G3335*), the alcohol production capability of the strain decreased significantly. The butanol and ethanol titers were 11.7 g/L and 1.1 g/L, respectively, a decrease of 17.5% and 33.3% relative to wild-type strain. Similar to the *adhE1* mutant, the *bdhB* mutant also accumulated lactate, with the maximal titer reaching 3.4 g/L. However, less lactate (0.6 g/L) was accumulated in the *bdhA* mutant compared with 1.0 g/L for control strain (Fig. 3).

As for acid production, the profile of butyrate production for the *bdhA* mutant was similar with the control. The mutant strain produced less acetate with approximately 0.7 g/L acetate accumulating in the fermentation broth (Fig. 3). The final pH of the mutant strain culture was higher than that of the control, presumably due to the lower residual acid levels. Especially in the later stage of fermentation, the pH continued to rise, which was coincident with an increase in ethanol production and decreased acetate production (Fig. 3). For the *bdhB* mutant, there was no remarkable difference in acetate production compared to the control strain, however, the strain accumulated three-fold more butyrate (1.8 g/L) than the control (Fig. 3), suggesting a weaker acid re-assimilation. The pH rebound observed for this mutant was not as significant as that for the wild-type strain (Fig. 3). This was also coincident with the poorer solventogenic performance of the mutant.

### Disruption of *SMB_P058* and *yqhD* leads to higher butanol/ethanol ratio however gives different metabolic profiles

**Figure 4:**
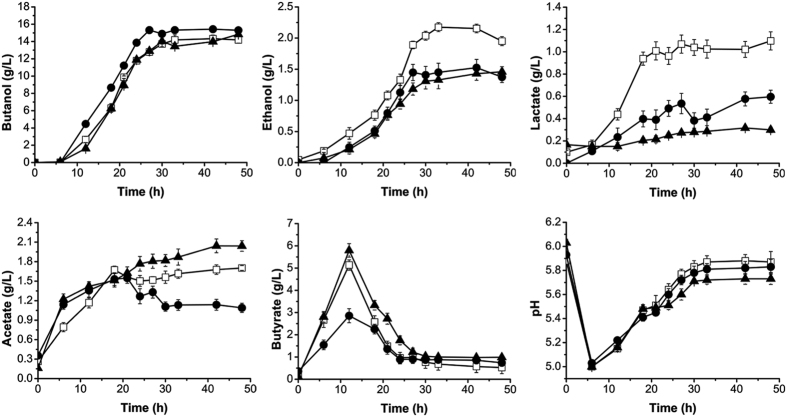
Fermentation profiles of *C. acetobutylicum* DSM 1731, *SMB_P058* mutant and *yqhD* mutant. White squares, wild-type; black triangles, *yqhD* mutant; black circles, *SMB_P058* mutant.

*SMB_P058* is carried by pSMBa, a megaplasmid of *C. acetobutylicum* DSM 1731, and is annotated as alcohol dehydrogenase. The mutant strain *C. acetobutylicum* DSM 1731 *SMB_P058*::int(150) showed different properties in butanol and ethanol production, producing more butanol (15.4 g/L) and less ethanol (1.52 g/L) than the wild-type (Fig. 4). Thus the butanol/ethanol ratio improved from 6.65:1 to 10.12:1 (Table 1). These data indicate that the enzyme product of gene *SMB_P058* favors biosynthesis of ethanol over butanol. *yqhD* on chromosome was also annotated as an alcohol dehydrogenase. The *yqhD* mutant strain produced less ethanol (1.46 g/L) than the control without a significant effect on butanol production (Fig. 4), which led to an increased butanol/ethanol ratio (10.17:1) (Table 1). We therefore propose that YqhD contributes to ethanol but not butanol production. These two mutant strains produced less lactate compared with control strain (Fig. 4), however, on other hand, these two strains had a higher butanol yield with 19.2% for *SMB_P058* mutant and 19.6% for *yqhD* mutant relative to the control (Table 1). Similar to the *adhE2* mutant, strain *SMB_P058*::int(150) accumulated less butyrate before acid assimilation with the peak value of butyrate production being much lower. We speculate that this profile is due to an improved ability to assimilate acid by *SMB_P058*::int(150). For *yqhD* mutant, more acetate (2.0 g/L) was accumulated compared with the control, reflected in the lower pH observed in the later stages of the fermentation (Fig. 4).

## Discussion

In *C. acetobutylicum*, several different aldehyde/alcohol dehydrogenases are involved in butanol and ethanol production, each with specific order of expression, expression level and substrate preference. In this work, our data show that the *adhE1* mutant almost lost the ability to produce alcohol during glucose batch fermentation. *adhE1* and *adhE2* located in the megaplasmid were the only two genes encoding aldehyde dehydrogenase in *C. acetobutylicum*. It has been known that AdhE2 is expressed only under alcohologenic conditions^7^,^20^,^21^,^22^,^23^ which were not employed here. AdhE1 is the only aldehyde dehydrogenase which provides aldehyde for alcohol production via other alcohol dehydrogenases. Expressing an aldehyde dehydrogenase from *C. beijerinckii* NCIMB 8052 in the *adhE1* mutant partly restored acetone and alcohol production (Figure S1). The transcription level of *adhE1* was also the highest among all the aldehyde/alcohol dehydrogenases in *C. acetobutylicum*^14^. These data indicated that the aldehyde dehydrogenase activity of *adhE1* could not be substituted by AdhE2, suggesting an essential role of AdhE1 in butanol and ethanol generation. This work also corroborates earlier work highlighting the primary role of aldehyde dehydrogenase activity of AdhE1 in *C. acetobutylicum*^24^.

AdhE2 is responsible for alcohol production only under alcohologenic conditions which can be defined as growth with high NADH/NAD^+^ ratio at neutral pH in glucose-limited cultures after addition of neutral red or methyl viologen, or in a more reductive culture medium like glucose-glycerol or glucose-glycerol-pyruvate^7^,^20^,^21^,^22^,^23^. A recent study showed that AdeE2 contributed almost 100% for butanol production under alcoholgenic conditions whilst contributing no greater than 9% under solventogenic conditions^19^. This suggests that the expression of *adhE2* is sensitive to culture conditions, which may be one reason why our results differ from those in a previous report where there was no change of fermentation products in *adhE2* mutant^25^. In this study, calcium carbonate was used as a buffering agent in the supplemented Clostridium basal medium (CBM) in an anaerobic cabinet^25^, whereas here we controlled the pH of the clostridial growth medium (CGM) at ≥5.0 by addition of ammonia to the anaerobic bioreactor (solventogenic condition). In solventogenic conditions, *adhE2* was transcribed at a much lower level than *adhE1*^14^. Perhaps the disruption of *adhE2* led to favorable substrate and redox equivalent availability for *adhE1*, the only enzyme in a adhE2 minus background that could simultaneously convert acetyl-CoA and butyryl-CoA to alcohol and provide substrate aldehyde for other alcohol dehydrogenases. These could therefore explain why butanol production increased in the *adhE2* mutant. On other hand, the synergistic effect of an increased acid reutilization and improved cell growth (Figure S2) could be another reason, however, the mechanism behind this is, so far, unclear.

Isoenzymes BdhB and BdhA showed very different effects on the metabolic profile of *C. acetobutylicum.* It was shown that BdhB was very important for butanol and ethanol production in *C. acetobutylicum* second to only AdhE1. Although the ability to produce alcohol was significantly impaired in the *bdhB* mutant, the butanol production of the *bdhA* mutant was similar to that of the control. This might result from a difference on the transcription level and substrate specificity of the two alcohol dehydrogenases. The transcription of *bdhA* occurs earlier than that of *bdhB*, however the final transcription level of *bdhB* is significantly higher than that of *bdhA*^8^,^14^. In terms of enzyme activity and substrate specificity: BdhA has only two-fold higher activity with butyraldehyde than with acetaldehyde, whereas BdhB has 46-fold higher activity^26^. Therefore, BdhA is thought to be functional in low-level butanol production, whereas massive and continuous butanol production can be brought about by BdhB^27^,^28^.

The fermentation profiles of the *SMB_P058* and *yqhD* mutants were highly unexpected. Both mutant strains accumulated less ethanol than the wild-type yet did not show loss of butanol production. These two genes correspond to *cap0059* and *cac3392* in *C. acetobutylicum* ATCC 824. Both were proposed to be alcohol dehydrogenases for butanol and ethanol production^9^,^10^,^29^. No previous experimental data exist for their specificity in butanol and ethanol formation. *SMB_P058* was annotated as an iron-containing alcohol dehydrogenase, however, compared with characterized alcohol dehydrogenases, this enzyme showed its highest identity (38%) with the AdhB from the ethanologenic bacterium *Zymomonas mobilis* ZM4. AdhB is an important enzyme for ethanol production in *Z. mobilis* and showed 100% activity towards acetaldehyde and no activity towards butyraldehyde^30^. The decreased ethanol production by the SMB_P058 mutant in the present work suggests SMB_P058 may be more specific for ethanol production than for butanol. YqhD was annotated as an NAD(P)H dependent butanol dehydrogenase^9^,^10^,^19^. This enzyme showed the highest identity (57%) with the experimentally characterized alcohol dehydrogenase YqhD from *E. coli*. Overexpression of *yqhD* in *E. coli* has previously shown to be more beneficial for synthesis of ethanol than butanol, and deletion of this gene leads to a reduction in ethanol formation without adverse effects on butanol formation^13^. These results are very similar to our own findings which also indicate that YqhD in *C. acetobutylicum* favors the synthesis of ethanol over butanol. These results suggest that SMB_P058 and YqhD are very promising candidates for reducing by-product ethanol levels; double deletion of these two genes may therefore lead to a highly optimal butanol-producing strain.

All six aldehyde/alcohol dehydrogenases in *C. acetobutylicum* are NAD(P)H dependent enzymes. These enzymes play an important role in balancing the cellular redox potential by synthesizing alcohol, regenerating NAD^+^ for glycolysis. The disruption of these genes may cause redistribution of redox potential. In this work, we found that (NADH dependent) lactate formation was an important outlet for maintaining redox balance in *C. acetobutylicum*. In the *adhE1* and *bdhB* mutant strains, increased lactate accumulation occurred following a decrease in alcohol production. There are four lactate dehydrogenases in *C. acetobutylicum* DSM 1731 with one in particular, *ldh1* (*SMB_G0272*), showing to be remarkably upregulated (36-fold) in the *adhE1* mutant strain (data not shown). This lactate dehydrogenase is therefore a promising candidate for further metabolic engineering work, to increase the downstream redox potential driving force and further promote butanol production in cells.

Disruption of aldehyde/alcohol dehydrogenases led to a redistribution of redox equivalents between different NAD(P)H dependent enzymes and resulted in an increase in butanol production. However, the increased level is not enough for industrial relevant applications. One reason may be the diversity of aldehyde/alcohol dehydrogenases (enzyme activity, expression level, expression order, and substrate specificity) in *C. acetobutylicum*. The redox equivalent driving force caused by a single gene disruption may not be strong enough to favor sufficient butanol formation. In *C. acetobutylicum*, hydrogen production is another crucial regulator of cell redox equivalents. Thus, combinational optimization of the aldehyde/alcohol dehydrogenases system, and lactate dehydrogenases system together with systematic engineering of the hydrogenases may be a way to realize homo-butanol fermentation in *C. acetobutylicum*.

## Materials and Methods

### Strains, plasmids and culture conditions

A list of bacterial strains and plasmids used in this study is presented in Table 2. *C. acetobutylicum* DSM 1731, as used in our previous studies^15^,^31^, was used as the wild-type strain. *E. coli* JM109 was used for vectors construction. *E. coli* TOP10 bearing the methylating plasmid pAN2 was used for methylation of plasmids. *E. coli* strains were grown aerobically at 37 °C with shaking at 220 rpm in liquid LB medium or on LB medium supplemented with 1.5% agar. *C. acetobutylicum* strains were grown anaerobically at 37 °C in reinforced clostridial medium (RCM)^32^. pMTL008^17^ was used as the backbone to construct different gene insertion plasmids. Ampicillin, erythromycin and chloromycetin were added at concentrations of 100, 50 and 30 μg/mL, respectively, when necessary. All strains were stored in 15% glycerol at −80 °C. Cell growth was monitored by measuring the absorbance at 600 nm (OD_600_) with a UV/Vis 2802PC spectrophotometer (Unico, NJ, USA).

### DNA manipulation

All primers used in this study are listed in Additional File1: Table S1. All DNA manipulations including PCR product purification, plasmids DNA purification and total genomic DNA extraction were performed using kits from E.Z.N.A (Omega Biotek Inc., Guangzhou, China). PCR polymerase, restriction enzymes and T4 ligase were obtained from New England Biolabs (Beijing, China), and used according to the manufacturer’s instructions. Before transformation into *C. acetobutylicum*, all plasmids were methylated in *E. coli* TOP10(pAN2) to protect them from the restriction system^33^.

### Construction of group II intron based gene insertion plasmids

To obtain alcohol dehydrogenase negative mutants, the group II intron based ClosTron system was used^16^,^34^. A computer algorithm^18^ was used for designing intron re-targeting primers (Supplementary file: Table S1). PCR was performed according to the Targetron Gene Knockout System Kit Protocol (http://www.sigmaaldrich.com). The generated 350-bp DNA fragment was ligated into the vector pMTL008^17^ after digestion with *Hind*III and *BsrG*I. Positive clones were screened using specific primers after transformation into *E. coli* JM109. Electrotransformation of *C. acetobutylicum* was performed according to the method developed by Mermelstein^35^. The transformants were first selected on RCM plates containing 30 μg/mL chloromycetin for 48 h. Colonies were then suspended in liquid RCM containing 30 μg/mL chloromycetin overnight. Cells (100 μL) were plated at different dilutions at RCM plates containing 50 μg/mL erythromycins. The positive mutants were selected by colony PCR with primers *gene*-1 and *gene*-2. The expected mutation resulted in an enlarged PCR product compared with that obtained from the wild-type. DNA sequencing was used to confirm the correct insertion of the intron into the target site.

### Southern blotting

To verify that only one copy of the intron was inserted into the genome, Southern blot hybridization was performed as described in previous work^17^ with slight modifications. Primers intron-probe-1 and intron-probe-2 were used to generate specific II intron probes with plasmid pMTL008 as the template. Genomic DNA of wild-type strain DSM 1731 and the mutants was digested with *Hind*III. No DNA fragment released from the wild-type strain hybridized to the probe. Whereas, restriction fragments of the expected size derived from the mutants gave positive bands on the hybridization membrane.

### Batch fermentation and analytical method

To analyze the effect of the aldehyde/alcohol dehydrogenases on cellular metabolites, especially ethanol and butanol production, batch fermentation of *C. acetobutylicum* DSM 1731 and the mutant strains was performed in CGM in BioFlo 110 fermenters (New Brunswick Scientific, Edison, NJ, USA), according to the fermentation method described in previous work^36^. Cell growth, glucose consumption, and the main fermentation products (butanol, ethanol, acetone, butyrate, acetate and lactate) were determined with the same methods used in previous work^36^.

## Additional Information

**How to cite this article**: Dai, Z. *et al*. Elucidating the contributions of multiple aldehyde/alcohol dehydrogenases to butanol and ethanol production in *Clostridium acetobutylicum.* *Sci. Rep.* **6**, 28189; doi: 10.1038/srep28189 (2016).

## Supplementary Material

Supplementary Information

## Figures and Tables

**Table 1:** Comparison of fermentation parameters of different strains.

Strains	Solvent yield	Butanol yield	Butanol/ethanol ratio
*C. acetobutylicum* DSM 1731	29.1%	18.2%	6.65:1
*C. acetobutylicum* DSM 1731 *adhE2*::int(846)	31.8%	20.3%	8.15:1
*C. acetobutylicum* DSM 1731 *bdhA*::int(133)	31.5%	19.0%	5.63:1
*C. acetobutylicum* DSM 1731 *SMB_P058*::int(150)	28.8%	19.2%	10.12:1
*C. acetobutylicum* DSM 1731 *yqhD*::int(405)	29.9%	19.6%	10.17:1

**Table 2:** Strains and plasmids used in this study.

Strains and plasmids	Relevant characteristics	Source or reference
Strains		
* C. acetobutylicum* DSM 1731	Wild type strain	DSMZ
* C. acetobutylicum* DSM 1731 *adhE1*::int(807)	The *adhE1* II intron insertion gene mutant of *C. acetobutylicum* DSM 1731	This study
* C. acetobutylicum* DSM 1731 *adhE2*::int(846)	The *adhE2* II intron insertion gene mutant of *C. acetobutylicum* DSM 1731	This study
* C. acetobutylicum* DSM 1731 *bdhA*::int(133)	The *bdhA* II intron insertion gene mutant of *C. acetobutylicum* DSM 1731	This study
* C. acetobutylicum* DSM 1731 *bdhB*::int(669)	The *bdhB* II intron insertion gene mutant of *C. acetobutylicum* DSM 1731	This study
* C. acetobutylicum* DSM 1731 *yqhD*::int(405)	The *yqhD* II intron insertion gene mutant of *C. acetobutylicum* DSM 1731	This study
* C. acetobutylicum* DSM 1731 *SMB_P058*::int(150)	The *SMB_P058* II intron insertion gene mutant of *C. acetobutylicum* DSM 1731	This study
* E. coli* JM109	*recA1 mcrB *+ *hsdR17*	Lab storage
* E. coli* TOP10	*mcrA Δ(mrr-hsdRMS-mcrBC) recA1* Used for plasmid methylation before transformed into *C. acetobutylicum*	Invitrogen
Plasmids		
* *pAN2	Ф*3tI*, *p15aori*, Tet^R^	16
* *pMTL008	Deprived from pMTL007*-cac824I71a*, replacement of the *lacI-traJ-oriT-fac* promoter fragment by *Pthl*	17
* *pMTL008-*adhE1*	*adhE1* gene II intron insertion inactivation plasmid	This study
* *pMTL008-*adhE2*	*adhE2* gene II intron insertion inactivation plasmid	This study
* *pMTL008-*bdhA*	*bdhA* gene II intron insertion inactivation plasmid	This study
* *pMTL008-*bdhB*	*bdhB* gene II intron insertion inactivation plasmid	This study
* *pMTL008-*yqhD*	*yqhD* gene II intron insertion inactivation plasmid	This study
* *pMTL008-*SMB_P058*	*SMB_P058* gene II intron insertion inactivation plasmid	This study

Abbreviations: Tet^R^ tetracycline resistance, Ф*3tI*, Ф3TI methyltransferase gene of *Bacillus subtilis* phage Ф3TI; *Pthl*, the promoter of thiolase gene from *C. acetobutylicum*; DSMZ, German Collection of Microorganisms and Cell Cultures, Braunschweig, Germany.

## References

[b1] Atsumi S. . Engineering the isobutanol biosynthetic pathway in *Escherichia coli* by comparison of three aldehyde reductase/alcohol dehydrogenase genes. Appl Microbiol Biotechnol 85, 651–657 (2010). 19609521 10.1007/s00253-009-2085-6 PMC2802489

[b2] Atsumi S. . Metabolic engineering of *Escherichia coli* for 1-butanol production. Metab Eng 10, 305–311 (2008). 17942358 10.1016/j.ymben.2007.08.003

[b3] Shen C. R. . Driving forces enable high-titer anaerobic 1-butanol synthesis in *Escherichia coli*. Appl Environ Microbiol 77, 2905–2915 (2011). 21398484 10.1128/AEM.03034-10 PMC3126405

[b4] Yim H. . Metabolic engineering of *Escherichia coli* for direct production of 1,4-butanediol. Nat. Chem. Biol. 7, 445–452 (2011). 21602812 10.1038/nchembio.580

[b5] Emptage M., Haynie S. L., Laffend L. A., Pucci J. P. & Whited G. Process for the biological production of 1, 3-propanediol with high titer. US patent 6, 514, 733 (2003).

[b6] Nair R. V., Bennett G. N. & Papoutsakis E. T. Molecular characterization of an aldehyde/alcohol dehydrogenase gene from *Clostridium acetobutylicum* ATCC 824. J Bacteriol 176, 871–885 (1994). 8300540 10.1128/jb.176.3.871-885.1994 PMC205125

[b7] Fontaine L. . Molecular characterization and transcriptional analysis of *adhE2*, the gene encoding the NADH-dependent aldehyde/alcohol dehydrogenase responsible for butanol production in alcohologenic cultures of *Clostridium acetobutylicum* ATCC 824. J Bacteriol 184, 821–830 (2002). 11790753 10.1128/JB.184.3.821-830.2002 PMC139506

[b8] Walter K. A., Bennett G. N. & Papoutsakis E. T. Molecular characterization of two *Clostridium acetobutylicum* ATCC 824 butanol dehydrogenase isozyme genes. J Bacteriol 174, 7149–7158 (1992). 1385386 10.1128/jb.174.22.7149-7158.1992 PMC207405

[b9] Nolling J. . Genome sequence and comparative analysis of the solvent-producing bacterium *Clostridium acetobutylicum*. J Bacteriol 183, 4823–4838 (2001). 11466286 10.1128/JB.183.16.4823-4838.2001 PMC99537

[b10] Bao G. . Complete genome sequence of *Clostridium acetobutylicum* DSM 1731, a solvent-producing strain with multireplicon genome architecture. J Bacteriol 193, 5007–5008 (2011). 21742891 10.1128/JB.05596-11 PMC3165653

[b11] Harris L. M., Blank L., Desai R. P., Welker N. E. & Papoutsakis E. T. Fermentation characterization and flux analysis of recombinant strains of *Clostridium acetobutylicum* with an inactivated *solR* gene. J Ind Microbiol Biotechnol 27, 322–328 (2001). 11781808 10.1038/sj.jim.7000191

[b12] Jang Y.-S. . Enhanced butanol production obtained by reinforcing the direct butanol-forming route in *Clostridium acetobutylicum*. mBio 3, e00314–00312 (2012). 23093384 10.1128/mBio.00314-12 PMC3482502

[b13] Dellomonaco C., Clomburg J. M., Miller E. N. & Gonzalez R. Engineered reversal of the beta-oxidation cycle for the synthesis of fuels and chemicals. Nature 476, 355–359 (2011). 21832992 10.1038/nature10333

[b14] Alsaker K. V. & Papoutsakis E. T. Transcriptional program of early sporulation and stationary-phase events in *Clostridium acetobutylicum*. J Bacteriol 187, 7103–7118 (2005). 16199581 10.1128/JB.187.20.7103-7118.2005 PMC1251621

[b15] Mao S. . Proteome reference map and comparative proteomic analysis between a wild type *Clostridium acetobutylicum* DSM 1731 and its mutant with enhanced butanol tolerance and butanol yield. J Proteome Res 9, 3046–3061 (2009). 20426490 10.1021/pr9012078

[b16] Heap J. T., Pennington O. J., Cartman S. T., Carter G. P. & Minton N. P. The ClosTron: a universal gene knock-out system for the genus *Clostridium*. J Microbiol Methods 70, 452–464 (2007). 17658189 10.1016/j.mimet.2007.05.021

[b17] Dong H. J., Zhang Y. P., Dai Z. J. & Li Y. Engineering *Clostridium* strain to accept unmethylated DNA. PLoS One 5, e9038 (2010). 20161730 10.1371/journal.pone.0009038 PMC2817722

[b18] Perutka J., Wang W. J., Goerlitz D. & Lambowitz A. M. Use of computer-designed group II introns to disrupt *Escherichia coli* DExH/D-box protein and DNA helicase genes. J Mol Biol 336, 421–439 (2004). 14757055 10.1016/j.jmb.2003.12.009

[b19] Yoo M. . A Quantitative System-Scale Characterization of the Metabolism of *Clostridium acetobutylicum*. MBio 6, e01808–01815 (2015). 26604256 10.1128/mBio.01808-15 PMC4669385

[b20] Girbal L., Vasconcelos I., Saintamans S. & Soucaille P. How neutral red modified carbon and electron flow in *Clostridium acetobutylicum* grown in chemostat culture at neutral pH. *FEMS* Microbiol Rev 16, 151–162 (1995).

[b21] Peguin S. & Soucaille P. Modulation of carbon and electron flow in *Clostridium acetobutylicum* by iron limitation and methyl viologen addition. Appl Environ Microbiol 61, 403–405 (1995). 16534918 10.1128/aem.61.1.403-405.1995 PMC1388339

[b22] Girbal L., Croux C., Vasconcelos I. & Soucaille P. Regulation of metabolic shifts in *Clostridium acetobutylicum* ATCC 824. *FEMS* Microbiol Rev 17, 287–297 (1995).

[b23] Honicke D., Janssen H., Grimmler C., Ehrenreich A. & Lutke-Eversloh T. Global transcriptional changes of *Clostridium acetobutylicum* cultures with increased butanol:acetone ratios. N Biotechnol 29, 485–493 (2012). 22285530 10.1016/j.nbt.2012.01.001

[b24] Nair R. V. & Papoutsakis E. T. Expression of plasmid-encoded *aad* in *Clostridium acetobutylicum* M5 restores vigorous butanol production. J. Bacteriol. 176, 5843–5846 (1994). 8083176 10.1128/jb.176.18.5843-5846.1994 PMC196790

[b25] Cooksley C. M. . Targeted mutagenesis of the *Clostridium acetobutylicum* acetone-butanol-ethanol fermentation pathway. Metab Eng 14, 630–641 (2012). 22982601 10.1016/j.ymben.2012.09.001

[b26] Welch R. W., Rudolph F. B. & Papoutsakis E. T. Purification and characterization of the NADH-dependent butanol dehydrogenase from *Clostridium acetobutylicum* ATCC 824. Arch Biochem Biophys 273, 309–318 (1989). 2673038 10.1016/0003-9861(89)90489-x

[b27] Durre P. . Solventogenic enzymes of *Clostridium acetobutylicum*: catalytic properties, genetic organization, and transcriptional regulation. *FEMS* Microbiol Rev 17, 251–262 (1995). 10.1111/j.1574-6976.1995.tb00209.x 7576767

[b28] Durre P. Fermentative butanol production: bulk chemical and biofuel. Ann N Y Acad Sci 1125, 353–362 (2008). 18378605 10.1196/annals.1419.009

[b29] Papoutsakis E. T. Engineering solventogenic clostridia. Curr Opin Biotechnol 19, 420–429 (2008). 18760360 10.1016/j.copbio.2008.08.003

[b30] Kinoshita S., Kakizono T., Kadota K., Das K. & Taguchi H. Purification of two alcohol dehydrogenases from *Zymomonas mobilis* and their properties. Appl Microbiol Biotechnol 22, 249–254 (1985).

[b31] Wang S. H. . Formic acid triggers the “acid crash” of acetone-butanol-ethanol fermentation by *Clostridium acetobutylicum*. Appl Environ Microbiol 77, 1674–1680 (2011). 21216898 10.1128/AEM.01835-10 PMC3067271

[b32] Hirsch A. & Grinsted E. Methods for the growth and enumeration of anaerobic spore-formers from cheese, with observations on the effect of nisin. J Dairy Res 21, 101–110 (1954).

[b33] Mermelstein L. D. & Papoutsakis E. T. *In vivo* methylation in *Escherichia coli* by the *Bacillus subtilis* phage phi 3T I methyltransferase to protect plasmids from restriction upon transformation of *Clostridium acetobutylicum* ATCC 824. Appl Environ Microbiol 59, 1077–1081 (1993). 8386500 10.1128/aem.59.4.1077-1081.1993 PMC202241

[b34] Heap J. T. . The ClosTron: Mutagenesis in *Clostridium* refined and streamlined. J Microbiol Methods 80, 49–55 (2010). 19891996 10.1016/j.mimet.2009.10.018

[b35] Mermelstein L. D., Welker N. E., Bennett G. N. & Papoutsakis E. T. Expression of cloned homologous fermentative genes in *Clostridium acetobutylicum* ATCC 824. Nat Biotechnol 10, 190–195 (1992). 10.1038/nbt0292-190 1368230

[b36] Dai Z. . Introducing a single secondary alcohol dehydrogenase into butanol-tolerant *Clostridium acetobutylicum* Rh8 switches ABE fermentation to high level IBE fermentation. Biotechnol Biofuels 5, 44 (2012). 22742819 10.1186/1754-6834-5-44 PMC3674747

