# Supplementary material for: Elucidating the contributions of multiple aldehyde/alcohol dehydrogenases to butanol and ethanol production in Clostridium acetobutylicum
Source: Sci Rep. 2016 Jun 20;6:28189. doi: 10.1038/srep28189 (PMC4913296; doi:10.1038/srep28189)
Supplement: Supplementary Information [file srep28189-s1.pdf]

Scientific Reports

Elucidating the contributions of multiple aldehyde/alcohol dehydrogenases to butanol and ethanol production in *Clostridium acetobutylicum*

Zongjie Dai[1,2,3], Hongjun Dong[1], Yanping Zhang[1*], Yin Li[1]

[1]CAS Key Laboratory of Microbial Physiological and Metabolic Engineering, Institute of Microbiology, Chinese Academy of Sciences, Beijing, 100101, China

[2]Department of Biochemistry and Molecular Biology, University of Science and Technology of China, Hefei, 230026, China

[3]Present address: Department of Biology and Biological Engineering, Chalmers University of Technology, Gothenburg, SE-412 96, Sweden

[*]Correspondence: zhangyp@im.ac.cn

Table S1 Primers used in this study

Primers	Sequence (5'-3')	Source
EBS Universal	CGAAATTAGAAACTTGCGTTCAGTAAAC	This study
007-R1	AGGGTATCCCCAGTTAGTGTTAAGTCTTGG	This study
adhE1-807/808s-IBS	AAAAAAGCTTATAATTATCCTTAGAAAG<br>AGGAGCTGTGCGCCCAGATAGGGTG	This study
adhE1-807/808s-EBS1d	CAGATTGTACAAATGTGGTGATAACAGATA<br>AGTCGGAGCTTATAACTTACCTTTCTTTGT	This study
adhE1-807/808s-EBS2	TGAACGCAAGTTTCTAATTTCGGTT<br>CTTTCTCGATAGAGGAAAGTGTCT	This study
adhE1-intron check	ATAATTATCCTTAATACTTGATGCA	This study
adhE1-1	ATGAAAGTCACAACAGTAAAGGAAT	This study
adhE1-2	TTAAGGTTGTTTTTTAAAACAATTT	This study
adhE2-846/847s-IBS	AAAAAAGCTTATAATTATCCTTAAAAAT<br>AAAAGAAGTGCGCCCAGATAGGGTG	This study
adhE2-846/847s-EBS1d	CAGATTGTACAAATGTGGTGATAACAGATA<br>AGTCAAAGAAACTAACTTACCTTTCTTTGT	This study
adhE2-846/847s-EBS2	TGAACGCAAGTTTCTAATTTCGGTTA<br>TTTTTCGATAGAGGAAAGTGTCT	This study
adhE2-intron check	TGTGGTGATAACAGATAAGTCAAAGAAAC	This study
adhE2-1	CGCGGATCCATGAAAGTTACAAATCAAAAAGAAC	This study
adhE2-2	CGCGGCGCCTTAAAATGATTTTATATAGATATCC	This study
bdhA-133/134s-IBS	AAAAAAGCTTATAATTATCCTTAGTATA<br>AAAAGGAGTGCGCCCAGATAGGGTG	This study
bdhA-133/134s-EBS1d	CAGATTGTACAAATGTGGTGATAACAGATA<br>AGTCAAAGGAACTAACTTACCTTTCTTTG	This study
bdhA-133/134s-EBS2	TGAACGCAAGTTTCTAATTTCGGTTTA<br>TACTCGATAGAGGAAAGTGTCT	This study

bdhA-intron check	GTGATAACAGATAAGTCAAAGGAAC	This study
bdhA-1	GAGTGCTTATAGTTTATGGCGGAGG	This study
bdhA-2	CATGTCCCATGTATCGCCATCATAA	This study
bdhB-669/670s-IBS	AAAAAAGCTTATAATTATCCTTATTA<br>TTAAGAACTGTGCGCCCAGATAGGGTG	This study
bdhB-669/670s-EBS1d	CAGATTGTACAAATGTGGTGATAACAGAT<br>AAGTCGAACTTGTAACTTACCTTTCTTTG	This study
bdhB-669/670s-EBS2	TGAACGCAAGTTTCTAATTTCGGTTAA<br>TAATCGATAGAGGAAAGTGTCT	This study
bdhB-intron check	GTGATAACAGATAAGTCAGAACTTG	This study
bdhB-1	GCAGCAGGAACAGCTGATATTATGA	This study
bdhB-2	CTACGCCGTGTGTTATGTCGTAATA	This study
cac3392-405/406s-IBS	AAAAAAGCTTATAATTATCCTTAGTGCC<br>ACTTGGAGTGCGCCCAGATAGGGTG	This study
cac3392-405/406s-EBS1d	CAGATTGTACAAATGTGGTGATAACAGATA<br>AGTCCTTGGAACTAACTTACCTTTCTTTG	This study
cac3392-405/406s-EBS2	TGAACGCAAGTTTCTAATTTCGGTTG<br>GCACTCGATAGAGGAAAGTGTCT	This study
cac3392-intron check	GTGATAACAGATAAGTCCTTGGAAC	This study
cac3392-1	GAATTTGGAGGAATTGAACCTAATC	This study
cac3392-2	CTCAAGCACATGAACAAATGTATCT	This study
cap0059-150/151s-IBS	AAAAAAGCTTATAATTATCCTTAAAAA<br>AGGTCTTAGTGCGCCCAGATAGGGTG	This study
cap0059-150/151s-EBS1d	CAGATTGTACAAATGTGGTGATAACAGA<br>TAAGTCGTCTTAGTTAACTTACCTTTCTTTG	This study
cap0059-150/151s-EBS2	TGAACGCAAGTTTCTAATTTCGGTTT<br>TTTTTCGATAGAGGAAAGTGTCT	This study
cap0059-intron check	GTGATAACAGATAAGTCGTCTTAGT	This study

cap0059-1	GGCTCAAAGACTTCTTCCTTGGCGT	This study
cap0059-2	CTTAGCGCAGTCCATTACAGAACCA	This study
Intron-Probe-1	GTTATCACCACATTTGTACAA	This study
Intron-Probe-2	ACGCGTCGCCACGTAATAAATATC	This study

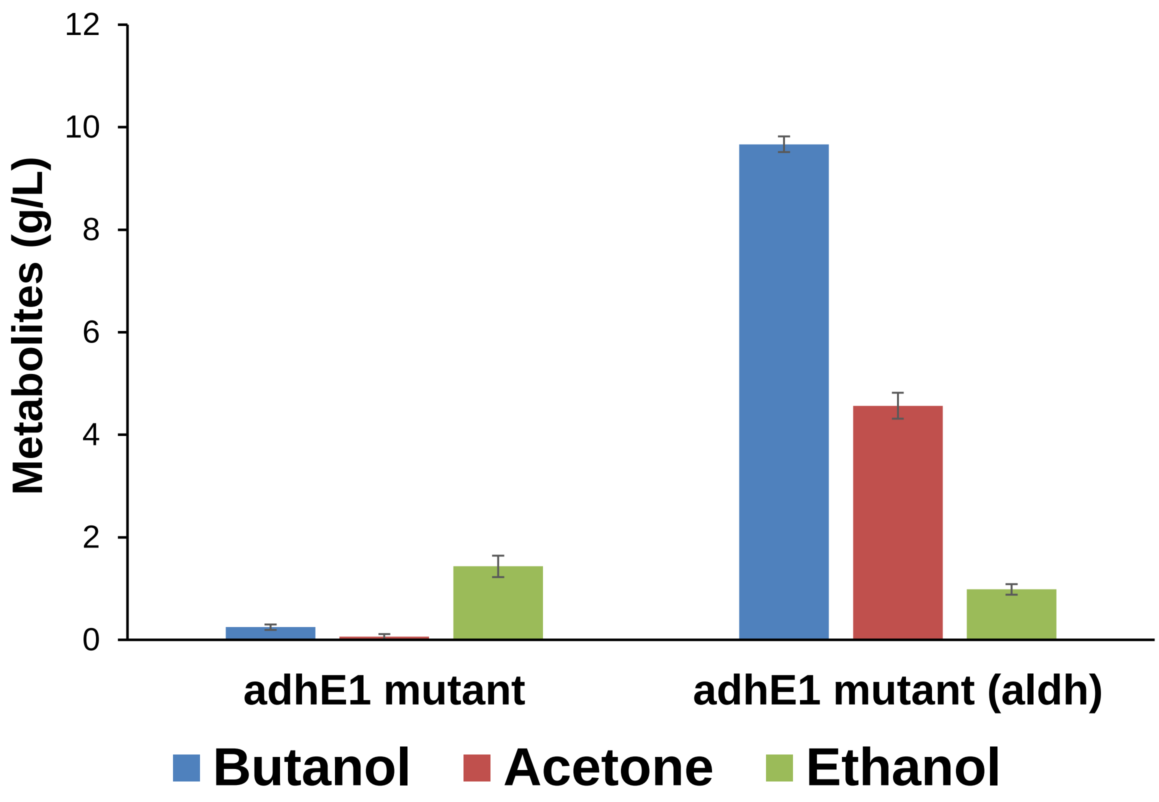

Figure S1. Solvent production profiles of *adhE1* mutant strain and *adhE1* mutant strain with aldehyde dehydrogenase (*aldh*)

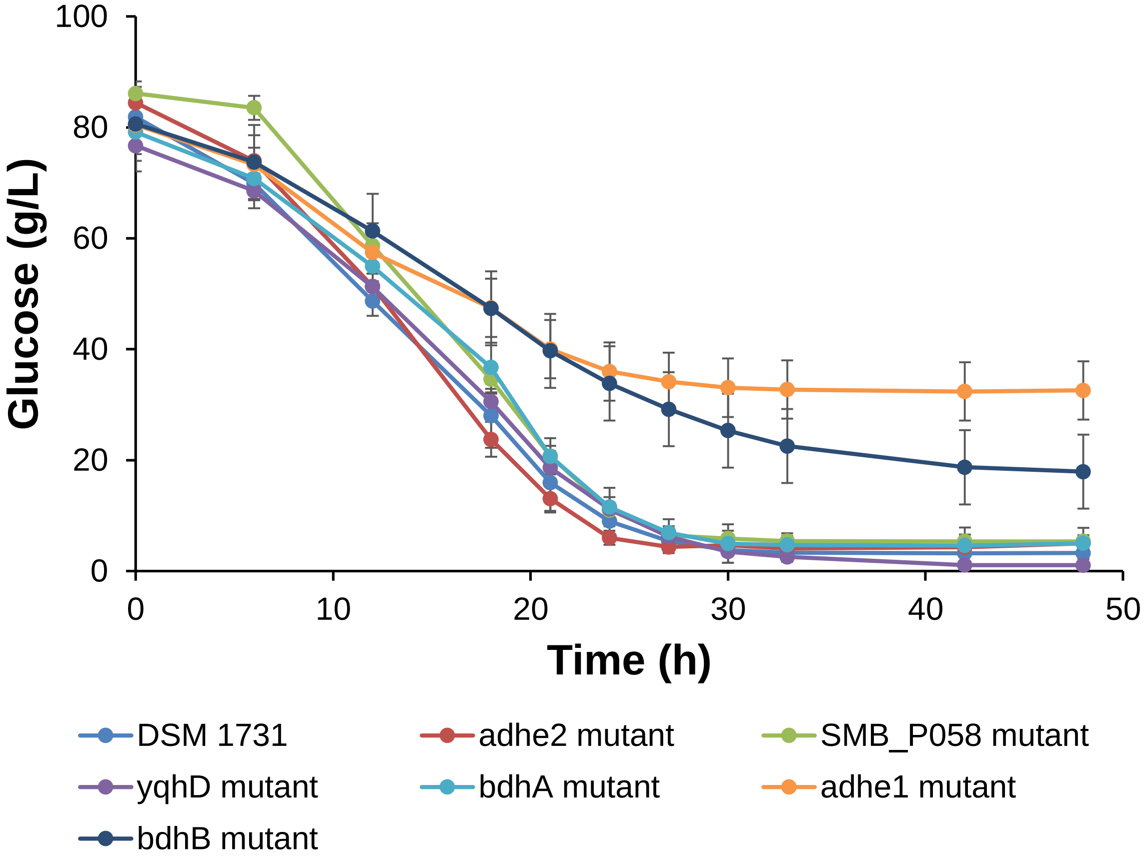
100
80
60
40
20
0
Glucose (g/L)
0
10
20
30
40
50
Time (h)
DSM 1731
adhe2 mutant
SMB_P058 mutant
yqhD mutant
bdhA mutant
adhe1 mutant
bdhB mutant

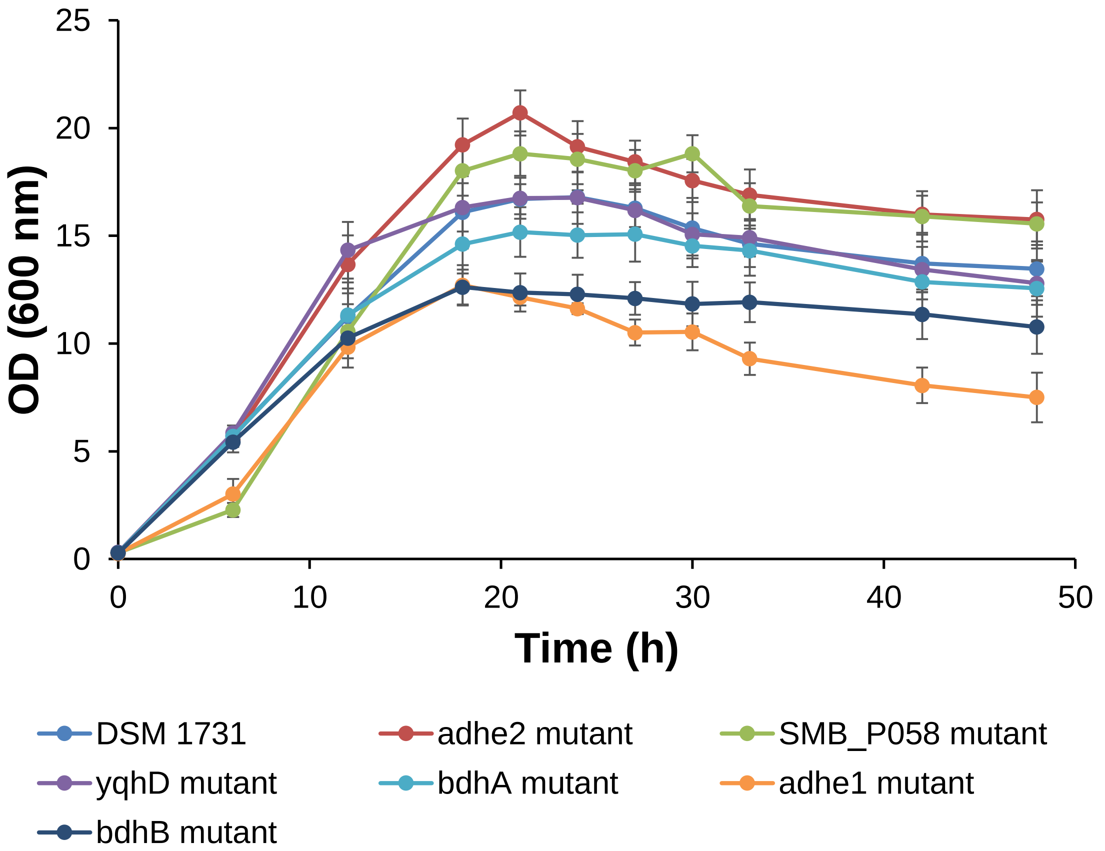
25
20
15
10
5
0
OD (600 nm)
0
10
20
30
40
50
Time (h)
DSM 1731
adhe2 mutant
SMB_P058 mutant
yqhD mutant
bdhA mutant
adhe1 mutant
bdhB mutant

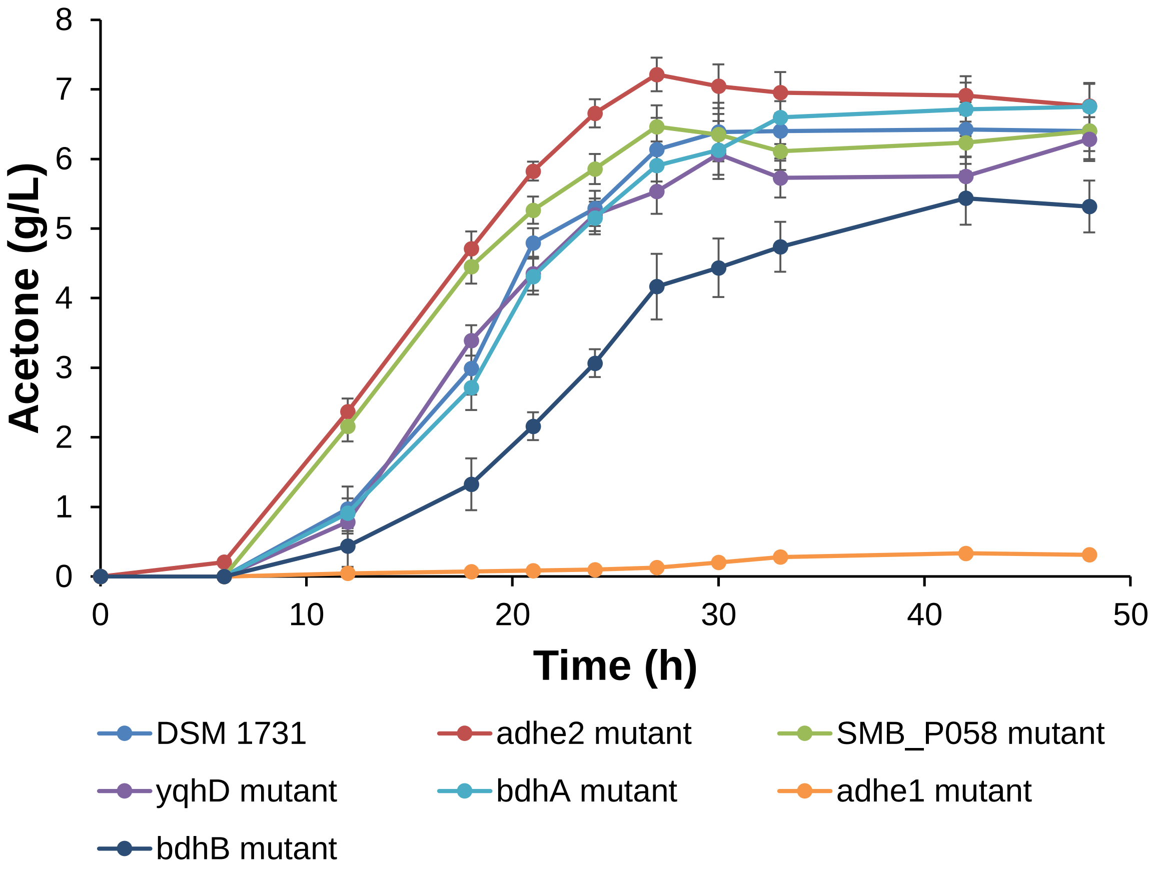

Figure S2. Glucose consumption, cell growth and acetone production of control strain and different mutant strains.
